# Sex chromosome aneuploidy in cytogenetic findings of referral patients from south of Iran

**Published:** 2012-03

**Authors:** Najmeh Jouyan, Elham Davoudi Dehaghani, Sara Senemar, Ashraf Shojaee, Hossein Mozdarani

**Affiliations:** 1 *Human Genetic Research Group, Iranian Academic Center for Education, Culture and Research, Fars Province Branch, Shiraz, Iran.*; 2 *Institute of Biochemistry and Biophysics, Tehran University, Tehran, Iran.*; 3 *Department of Medical Genetics, Faculty of Medical Sciences, Tarbiat Modares University, Tehran, Iran.*

**Keywords:** *Sex chromosome abnormality*, *Disorders of sexual development*, *South of Iran*, *Infertility*, *Cytogenetic*

## Abstract

**Background:** Chromosome abnormality (CA) including Sex chromosomes abnormality (SCAs) is one of the most important causes of disordered sexual development and infertility. SCAs formed by numerical or structural alteration in X and Y chromosomes, are the most frequently CA encountered at both prenatal diagnosis and at birth.

**Objective:** This study describes cytogenetic findings of cases suspected with CA referred for cytogenetic study.

**Materials and Methods:** Blood samples of 4151 patients referred for cytogenetic analysis were cultured for chromosome preparation. Karyotypes were prepared for all samples and G-Banded chromosomes were analyzed using x100 objective lens. Sex chromosome aneuploidy cases were analyzed and categorized in two groups of Turners and Klinefelter’s syndrome (KFS).

**Results:** Out of 230 (5.54%) cases with chromosomally abnormal karyotype, 122 (30%) cases suspected of sexual disorder showed SCA including 46% Turner’s syndrome, 46% KFS and the remaining other sex chromosome abnormalities. The frequency of classic and mosaic form of Turner’s syndrome was 33% and 67%, this was 55% and 45% for KFS, respectively.

**Conclusion:** This study shows a relatively high sex chromosome abnormality in this region and provides cytogenetic data to assist clinicians and genetic counselors to determine the priority of requesting cytogenetic study. Differences between results from various reports can be due to different genetic background or ethnicity.

## Introduction

Sex chromosome abnormalities (SCAs) are the most frequently occurring chromosomal abnormalities encountered at both prenatal diagnosis and at birth and are one of the most important causes of disorders of sexual development (1). 

Human infertility is closely linked to chromosomal abnormalities (CA) (2). Of those, sex chromosome aneuploidy was the most common (9%) and Klinefelter’s syndrome (KFS) was the most frequent sex chromosome anomaly in males with azoospermia (about 14% of cases with azoospermia) (3, 4). About the frequency of chromosomal abnormalities in female infertility there is contradiction (estimated in about 5%) (5). In the female partners of infertile couples undergoing the Intra Cytoplasmic Sperm Injection (ICSI), Intrauterine Insemination (IUI) or In-vitro Fertilization (IVF) procedures, CA rang from 1.1% to 9.8%; but it is more prevalent in sexual development problems for instance about 30% of primary amenorrhea are caused by Turner syndrome (6). 

Sex chromosome imbalance has a much less deleterious effect on the phenotype than dose autosomal aneuploidy. Historically, many affected individuals remained undiagnosed throughout their lifetimes due to generally mild and variable effects of the aneuploidy (7). 

The increased awareness of the importance of chromosomal abnormalities as a cause of primary or secondary amenorrhea in women, feminine distribution of adipose tissue, absence or decreased facial and pubic hair, small hyalinized testes and small penis in men and delayed pubertal development and/or infertility in both men and women has generated an increased demand of cytogenetic studies (8). The recent advances in cytogenetic techniques have provided a valuable means for modern medicine to recognize many chromosomal disorders that otherwise would have been missed (9). The introduction of the banding techniques in to cytogenetic has been regarded as a significant step in the identification of chromosomal anomalies which gave insight to many of the health problems (10).

In this study, we determined the frequency of chromosomal aberration among the different groups of referrals suspected of sex chromosomal abnormalities. It provides a foundation for regional cytogenetic data for the first time in southern part of Iran. It also shows the accordance rate between results obtained from cytogenetic study and diagnosing by clinical features which highlights the importance of Giemsa banding for correct identification of a variety of reproductive problems. In addition, we compared these results with those reported in similar studies to find probable differences.

## Materials and methods

In our retrospective study, over a period of 9 years from 2000-2009, a total of 4151 subjects suspected of chromosome anomalies were studied. These individuals presented the clinical features of KFS, Turner’s syndrome (TS), primary amenorrhea (PA), secondary amenorrhea (SA), sexual ambiguity, infertility, failure to thrive and recurrent abortion, Down’s syndrome and other types of mental retardation and some of them referred for premarriage cytogenetic tests. 

These cases were referred by physicians or consolers from different medical centers throughout Fars province and even other south provinces to genetic laboratory of Iranian Academic Center for Education, Culture and Research (ACECR), Fars province Branch. Of them 137, 354 and 30 cases suspected of KFS, TS and sexual ambiguity respectively and 180 cases were suspected of Down's syndrome. Exact and comprehensive clinical features of patients for suspicion and pattern of other referrals hadn't been documented. 

In this study we focused on sex chromosome abnormality. Heparinized peripheral blood taken from patients were cultured in RPMI 1640 medium (Gibco) supplemented with 15% Fetal Bovine Serum (FBS, Gibco), 100 µg/ml Penicillin and 100 µg/ml Streptomycin. 0.1ml of phytohemaglutinin (Sigma) was added to the cultures to initiate the cell cycle in lymphocytes. 

Cells were left in 37ºC CO_2_ (5%) incubator in a humid atmosphere for 72 hours. Harvesting and Chromosomal preparations were made according to standard methods (11). All chromosome preparations were G-banded according to the Seabright’s method (12). A minimum of 20 metaphases were scored in each case. In cases where mosaicism was detected, metaphases up to 50 were analyzed for numerical abnormalities and the best among them were karyotyped by Karyo imaging software (Italy).

**Statistical analysis**

The relative frequency of each diagnostic group was calculated, and the percentage of abnormal cases and the distribution of the numerical abnormalities were determined in each group using SPSS (version 16.5). The frequencies were compared to similar studies using the Z-test for comparison of two frequencies with unequal variance.

## Results

The percentage of chromosome abnormalities among all individuals referred to genetic lab of ACECR was 5.54%. (The reason for patients’ reference wasn’t mentioned separately). The autosomal chromosome abnormality was identified in 108 cases and the sex chromosome abnormalities in 122 cases with a frequency of 46%, including cases with KFS (46%), Turner’s syndrome (46%) and sexual ambiguity with XX/XY chimer karyotype (8%), among whom there were 2 cases with male socially sex and 6 cases with female socially sex. 

The average age of the female patients with Turner's syndrome was 20.8±5.77 (2-35 years) and it was 32.6±7.6 (23-49 years) for male patients with KFS. Among 56 cases with KFS, 31 cases showed 47/XXY karyotype, while 25 cases showed 46XY/47XXY mosaic chromosome compliment and out of 57 cases detected for Turner's syndrome, 19 cases were classic Turner with 45, X karyotype and 38 cases were mosaic Turner with 45, X/46XX karyotype. Table I shows the percentage of chromosome abnormalities detected among 122 referral subjects with disorder of sexual development.

**Table I:** Distribution of numerical sex chromosome abnormality and their classic and mosaic forms of them

**Type of disorder**	**Type of chromosome abnormality**	**Number of patients**	**Total**
Klinefelter’s syndrome	47, XXY 47,XXY / 46,XY	31 25	56
Turner’s syndrome	45,X 45,X / 45,XX	19 38	57
Sexual ambiguity	46,XX / 46,XY	9	9

**Table II:** Frequency of classic and mosaic form of Turner syndrome in some countries

**Country**	**Classic turner ** **(%)**	**Mosaic turner ** **(%)**	**Other ** **(%)**	**References**
Italy	50	35-40	10-15	41
Brazil	28.6	53	17.9	22
Tunisia	32	47	21	36
Denmark	45	15	40	35
Kuwait	63	22	15	39
Korea	2.1	50.8	10	18
Singapore	57.1	-	-	40
Minnesota	42	48	10	37
This study	34	66	-	-

**Table III:** Frequency of classic and mosaic form of Klinefelter’s syndrome in some countries

**Country**	**Classic Klinefelter (%)**	**Mosaic Klinefelter (%)**	**References**
Brazil	20	80	22
Denmark	89.7	6	35
India	80	20	45
Korea	86.4	-	18
Tunisia	66.6	33.4	44
This study	56	34	-

## Discussion

**Chromosome abnormality**

Chromosome abnormalities are important causes of lack of development in secondary sexual characteristics, delayed pubertal, miscarriage, infertility, etc. (13, 14). The identification of numerical and structural chromosome abnormalities by routine and high resolution cytogenetic studies plays an important role in the diagnosis and treatment of various diseases. In this study we evaluated patients referred for cytogenetic analysis, which 5.54% show chromosome abnormality. 

This result is similar to some other similar studies-with same methodology and unselected patients- such as the studies of Brum and Kumar *et al* which have reported 5.5% and 3.8% chromosome abnormality in patients suspected of chromosome anomalies (15, 16), but it is less than that observed in India (16.6%), Pars genetic lab of Tehran (15%), and Korea (17.5% and 15.3%) (10, 17-19). Santos *et al*, Singh and Duarte *et al* have reported the higher frequency of chromosome abnormality in their investigations (28.6%, 28.8% and 29.3% respectively) (20-22).

These differences can be resulted from employed complementary methods and type of them in various genetic labs. Structural aberrations in chromosomes such as small deletions, duplications and some translocations were not detected by common G-banding method, applying other advanced methods like high resolution and FISH improve accuracy of recognition, so increase recognized chromosomal abnormality (8). 

For instance, in a study mosaicism was detected in 7 out of 19 patients (37%) previously thought to be only a single 45,X cell line (23). In addition, we should know that cytogenetic study can be done due to two purposes, first as a primary study before other expensive or time-consuming tests and second, to ensure of non-cytogenetic basis of the disorder. 

So according to referral criteria, it is expected variation in chromosomal abnormality frequency in different genetic labs. However to avoid unnecessary referrals, conferring to experienced physicians prior to genetic studies is suggested (9).

Also this study shows that it shouldn't overlook submission of complementary test, improvement quality of methods and applying experienced experts. 

**Frequency of sex chromosomes abnormality (SCAs) compared to autosomal chromosome abnormality (ACA)**

Approximately, 1/400 newborns has SCA, making SCA twice as common at birth as trisomy 21 (24-26). In this study, of 230 abnormal karyotype out of 4151 karyotype analyzed, 122 (53%) cases showed sex chromosome abnormality including Turner’s syndrome, KFS and ambiguous abnormality and 108 (47%) cases showed autosomal chromosome abnormality including Down’s syndrome, Patau’s syndrome, Edward’s syndrome, etc. These results can show that aneuploidy involving the sex chromosomes is more common (but not significantly) than autosomal aneuploidy at least in this region. Of these syndromes, the highest referrals were for suspicion of TS and KFS (73.2%) following by Down syndrome (26.8%). 

However, we should be cautions to conclude because the difference between SAC and ACA frequency is not significant and can be varied according to individuals’ referral to Genetic labs. The rate of SCA in this study was lower than what was expected according to references. This could be due to this fact that SCAs are less deleterious than ACA and a lot of these problems such as Turner’s syndrome and KFS may only appear at puberty or later because of infertility or not detected at all. 

For example while KFS is the most common sex chromosome abnormality seen in infertile men (1, 27), about two-thirds of males with this syndrome are never detected (28) and other sexual abnormalities like XXX or XYY syndromes have such a mild symptoms that are out of clinical notice and/or a lot of individuals are unknown of their problems and almost don't refer to any genetic lab. Therefor increasing general knowledge about chromosome disorders should be considered.

Our data is different with others: In a study on 916 cases in Brazil 83.6 % ACA and 16.4% SCA has been reported (22). In two other studies in Korea 66.7% and 73% patients showed ACA against 33.3% and 26.9% SCA (18, 19). These differences can be related to different referral of people to labs depend on regional culture, knowledge and believes and even access to genetics labs. 

As well, it can attribute to genetic or environmental background which should be surveyed. In addition we find that 30% of patients suspected of TS or KFS had 45, X or 47, XXY karyotype and 55% of patients suspected of ACA had 47, XX, +21 or 47, XX, +21. This significant difference reflects difficulty of diagnosis in sexual disordered compare to autosome disorders also efficiency and importance of cytogenetic analysis to detect SCAs.

**Klinefelter and Turner syndromes**

Both KFS and Turner syndromes are compatible with life and according to statistical data, KFS is more prevalent at birth than Turner syndrome (7). Here KFS frequency diagnosed by karyotyping is approximately equal with Turner’s syndrome (Table I). These results are different from the frequency and prevalence of these syndromes; it can be due to more sensitivity of sexual disorders in girls and women or clearer feature to diagnose Turner's syndrome or less referral of men patients to labs compared to women. 

In spite of this fact that chromosomal abnormalities are more prevalent in infertile men (6), because of personal and cultural issues some men don’t accept probability of infertility of them and prevent doing any clinical or genetic tests. In addition, the results can be explained by notice to different rate of mortality in KFS and TS in various areas. In two cohort studies in Great Britain, it was shown that mortality in men with KFS had been higher than those in women with TS, so a great number of patients with KFS remain undiagnosed (29, 30).

Our data are close to those in a research in India (59 % TS and 41.3% KFS) and in Korea (58.7% TS and 41.3% KFS) (14, 18). But they are far from those detected in Brazil (79.5% TS and 11.4% KFS) (22).This can be due to different referral of patients to the labs or different genetic and environmental background.

In present study 47% of cases suspected of having KFS, were diagnosed as KFS and 24% cases suspected of having TS were diagnosed of as TS. These percentages demonstrate the importance of cytogenetic evaluation in patinas that are clinically abnormal. But the discrepancy observed between diagnosis through features and cytogenetic analysis especially in productivity problem of women, conduct physicians and patients to follow other diagnostic tests.

**Classic and mosaic forms of Klinefeleter and Turner syndromes**

In our study, classic Turner (45, X) was observed in 34% of cases whereas mosaic form (45, X/46XX) was found in 66% of the patients. These data show that Turner’s syndrome in mosaic status condition is more compatible with life than pure Turner’s syndrome (7). Some researchers believe that all live born females with Turner’s syndrome have a cell line containing two sex chromosomes that may be present at a low level of mosaism (31). 

These results correlate well with previous reports. In Iran, mosaic TS cases refereed to Endocrine and Metabolic Research Center in Isfahan University of Medical Sciences, Genetic Center of Urmieye University and Department of Medical Genetic of Mashhad University were 37.5%, 41.7% and 19%, respectively (32-34) which also are similar to those of other countries like America (36%) Denmark (45%), Tunisia (32%), Minnesota (42%), Brazil (28.6%) and Czechoslovakia (24%) (22, 23, 35-38). In Kuwait and Singapore, the classic form was prevalent with a frequency of 57.1% and 74.3% respectively (39, 40). 

As we know, approximately 50% of the Turners patients have a 45, X karyotype, with no second sex chromosome, either X or Y and 5-10 % have a duplication (isochromosome) of the long arm of one X/46, X, i(Xq). Most of the remaining cases are involved in mosaic form (41). Up to 5% of Turners are fertile and the likelihood to have follicles in their ovaries is highest among mosaic Turners syndrome girls, so finding 46, XX line in these patients could offer hope toward natural pregnancies by receiving hormone replacement therapy (42, 43).

About 80% of patients with 47, XXY bear a congenital numerical chromosome aberration. The other 20% are represented either by 47, XXY/46, XY mosaics or higher-grade sex chromosomal aneuploidy or structurally abnormal X chromosomes (45). Here in cases with KFS, we found 56% of classic form (47xxy) and 34% of mosaism. These frequencies are close to those reported in Tunisia by Abdelmoula *et al* 66.6% and 33.3% respectively (44). Frequency of classic KFS observed in Iran (Pars Hospital of Tehran) (79%), Denmark (89.7), India (80) and Korea (86.4) was more than our data (17, 19, 36, 45). 

All studies report the classic form more than the mosaic form, expect a study in Brazil that reported: 20% for classic forms against 80% for mosaic form (22). The detection of a possible low grade mosaicism in peripheral lymphocytes in KFS patients implies that KFS patients may have germ cells with normal 46, XY content in their testis which is a good sign to productivity by operation and other techniques (47)

The difference in the frequencies of classic and mosaic form of KFS among mentioned studies could reflect variations in the criteria for inclusion of patients; however, we should consider the fact that reference of individuals to genetic labs can be different in various areas depending on people's information and the frequency of disease in the society based on genetic or environmental factors. 

In addition, the number of patients investigated is so important, the more the individuals studied, the more the data are close to reality. Our data showing 56 karyotypes of KFS patients is similar to a study done in India (53 abnormal karyotype for KFS) (14) and our results about mosaism and classic from of KFS were similar but in Brazil they had 10 cases with KFS (22) and their results were different from what mentioned above.

## Conclusion

In conclusion the process of genetic counseling for sex chromosomal abnormalities is complex. Cytogenetic analysis is one of the most useful approaches to investigate the individuals with productivity or sexual problems of unknown origin to confirm the clinical diagnose in patients with a known cytogenetic syndrome or reject the chromosomal abnormality. Relative low accordance between clinical diagnosis of sexual disorders and results obtained from karyotyping in this study shows the importance of cytogenetic analysis for correct diagnosis of the disease. 

In addition with the advent of new molecular cytogenetic techniques such as fluorescent in situ hybridization (FISH), it is possible to detect these abnormalities in interphase cells accurately. Data obtained from these studies provide a foundation for regional cytogenetic data library to assist clinicians and genetic counselors determine the priority of requesting cytogenetic study. The discrepancies in the frequencies of cytogenetic abnormalities among the different investigations arise necessity of more studies to suggest reasons and following solution.
